# UGT2B7 gene polymorphism and linkage disequilibrium in pediatric epileptic patients and their influence on sodium valproate monotherapy: A cohort study

**DOI:** 10.3389/fphar.2022.911827

**Published:** 2022-09-09

**Authors:** Sachidananda Adiga, Nandit PB, Usha Adiga, Vijaya Shenoy

**Affiliations:** ^1^ Professor, Dept of Pharmacology, Biochemistry, Pediatrics K. S. Hegde Medical Academy, Nitte (Deemed to Be University), Mangalore, India; ^2^ Regional Medical Advisor, Rivaara Lab, Bangalore, India

**Keywords:** pediatric epilepsy, genetic polymorphism, sodium valproate, clinical outcome, linkage disequilibrium

## Abstract

**Background:** Uridine 5′-diphospho glucuronosyl transferase (UGT) is the main enzyme responsible for the glucuronide conjugation, the principal metabolic pathway of sodium valproate. The objective of the study was to explore if there was an association between the UGT2B7 genetic polymorphism and clinical efficacy and safety in paediatric epileptic patients on sodium valproate monotherapy.

**Methods and materials:** The cohort study included 100 pediatric epileptic patients aged 2–18 years who had been on sodium valproate monotherapy for at least 1 month. PCR-RFLP was carried out to assess the genetic polymorphism patterns of UGT2B7 (C161T, A268G, G211T). Based on the extent of seizure control throughout the 1-year follow-up, clinical outcome was assessed in terms of responders and non-responders. Hepatic, renal, and other lab parameters were assayed to determine safety. The SNPstat web software was used to calculate linkage disequilibrium.

**Results:** Out of 100 patients, CC (38%), CT (43%), TT (19%) pattern was observed in UGT2B7 (C161T) gene, AA (15%), AG (39%), GG (46%) in (A268G) gene and GG (80%), GT (18%), TT (02%) in (G211T) gene. There was no statistical difference in clinical outcome with distinct UGT2B7 genetic polymorphism patterns, according to the findings. With low D′ and R2 values, linkage disequilibrium between alleles was statistically insignificant. However, the associations of C161T and G211T with treatment response were significant (*p* = 0.014) in determining treatment response.

**Conclusion:** Our findings show that the genetic variation of UGT2B7 had no bearing on the clinical outcome of epilepsy. Gene interactions, on the other hand, had an impact on treatment response.

## Introduction

Epilepsy is a neurological illness that includes a variety of seizure types and epilepsy syndromes that are heterogeneous and complex brain disorders (Berg et al., 2010; Tejada et al., 2013). India’s overall epilepsy prevalence is estimated to be 3–11.9 per 1,000 people (Amudhan et al., 2015). After a patient has had unprovoked seizures, paediatricians and neurologists frequently prescribe long-term, anti-epileptic drugs (AEDs) (Cockerell et al., 1997). About 60–70% of newly treated patients have satisfactory seizure control (Sillanpaa and Schmidt, 2006) while the remaining 30% of patients will have uncontrolled epilepsy with recurrent seizures, adverse effects, and a significantly increased risk of mortality and morbidity if they are treated with AEDs for a long time (Sillanpaa and Schmidt, 2006; Tejada et al., 2013). The goal of treating epilepsy in children is to prevent seizures for 2 years with an anti-epileptic drug (AED) that has minimal side effects, so that the drug can be gradually taken off.

Pharmacogenetics is the study of genetic variants that impact drug metabolism, pharmacological targets, or disease pathways, resulting in variable responses to drugs in terms of efficacy or side effects. As a result, it is critical to look into the function of genetic polymorphisms in drug metabolism and antiepileptic drug effects.

Rationale: Sodium valproate is a widely used antiepileptic medication with a broad spectrum of action. Uridine-5′-diphosphate glucuronosyl transferases catalyse glucuronidation, a key route of VPA (UGTs). Changes in sodium valproate metabolism may be caused by polymorphisms in genes encoding UGT enzymes. Changes in sodium valproate’s glucuronidation rate can affect the drug’s blood level, resulting in either insufficient plasma concentrations that impair therapy effectiveness or elevated plasma concentrations that cause toxicity (Chatzistefanidis et al., 2012). The glucuronidation of sodium valproate is aided by polymorphisms in the rs7439366, rs12233719, and rs145725367 genes (Chatzistefanidis et al., 2012). The functional impacts of SNPs of UGT2B7 was investigated using computational prediction tools (Polyphen analysis and SIFT analysis). Three SNPs (rs12233719, rs7668258, rs7662029 were selected for the wet lab analysis. Although the selected variants were tolerant, studies have shown the significant role of those SNPs in different populations.

Novelty: UGT2B7 polymorphisms have previously been studied in Chinese and Japanese populations. The majority of investigations have focused on determining the pattern of genetic polymorphism and its impact on a patient’s serum concentration. However, there is very little evidence about the effect of sodium valproate on the UGT2B7 polymorphism and its relationship to clinical outcome in the Indian population.

Assessing patterns of linkage disequilibrium (LD) of single nucleotide polymorphisms (SNPs) has become an essential aspect of both evolutionary biology and medical genetics. Within a population, LD occurs when two alleles at different loci are genetically related and show non-random association in the same chromosome. Many factors influence LD, including selection, genetic recombination rate, mutation rate, genetic drift, mating system, population structure, and genetic linkage. Because the range of potential values relies on the frequencies of the alleles it refers to, the coefficient of linkage disequilibrium (D) is not necessarily a practical measure of linkage disequilibrium.

## Objective

The objective of the study was to explore the association between the UGT2B7 gene polymorphisms and clinical efficacy and safety in pediatric epileptic patients on sodium valproate monotherapy, as well as the linkage disequilibrium between the SNPs of UGT2B7 gene.

## Methodology

The observational cohort study was conducted from February 2018 to February 2020. Hundred pediatric epileptic patients of South Indian descent were included in the study which was conducted at the Justice K S Hegde Charitable Hospital in Mangalore, India, and the Central Research Laboratory of the K S Hegde Medical Academy in Mangalore.

### Inclusion criteria

Epileptic patients of 2–18 years of age were diagnosed clinically by EEG, patients on a stable dose of 20 mg/kg/day sodium valproate on a frequency of twice/thrice a day for the past 1 month.

Exclusion Criteria: Patients who were already on treatment with any other antiepileptic drugs or drugs which induce or inhibit enzymatic action of sodium valproate metabolism. History or evidence of hepatitis or impaired renal functions.

The research protocol was approved by Central Ethics committee of NITTE (Deemed to be University) (Approval number: NU/CEC/2018/0174 dated 19-01-2018, amendment in the proposal was approved by NU/CEC/2018/08 dated 26-11-2018, and approval to have house visit and telephonic data collection about the clinical pattern seizure control, recurrence and patient compliance (NU/CEC/2020/0300 dated 10-7-2020).

The research process was briefly explained orally in simple words in their native language for children in the age group of 7–12 years along with written consent from their parents. The written assent was obtained from children of 12–18 years and written consent was obtained from the participants parents in the presence of a witness (not related to patient and research team). The written consent was obtained from parents of eligible participants who are less than 6 years in the presence of a witness as mentioned above. The mentioned are the guidelines given by Indian Council of Medical Research for enrolling the participants for biomedical research (2016).

Once the patient met the requirements, 4 mL of whole blood were collected aseptically during the 6-months follow-up appointment. 2 ml of EDTA whole blood was preserved at -80°C for genetic polymorphism study, and 2 ml of whole blood in a plain vial was centrifuged to obtain serum and then stored at -80°C for biochemical parameters analysis.

At the time of enrolment and at 6 months, liver function tests (Albumin, total protein, total, direct bilirubin, SGOT, SGPT, ALP), renal function tests (Blood urea, serum creatinine), platelet count, and serum amylase were estimated.

### Genotyping

Blood sample was centrifuged with RBC lysing solution (NH4Cl, KHCO3, Na2. EDTA). The residual RBC lysate was suspended with a cell lysing solution (50 mM Tris HCl, 50 mM EDTA, 10 mM NaCl, 1% SDS) and the cell lysates was digested overnight followed by the addition of protein precipitating solution. After centrifugation (3000 RPM, 10 min), the supernatant was collected in the 2% isopropanol and centrifuged again. The pellet which consists of DNA was washed with 70% ethanol and the DNA was allowed to precipitate. The precipitated DNA was transferred to the vials containing 50 µl of TE buffer (pH 7.5). The quantity of the DNA isolated was determined using a nanodrop spectrophotometer (Eppendorf) at 260 nm. The purity of the DNA sample was calculated by the ratio of 260/280 nm. Quantified DNA was sealed and stored at −20°C until further analysis.

The UGT2B7 polymorphisms at C161T, G211T, A268G were analyzed by the Polymerase Chain Reaction–Restriction Fragment Length Polymorphism (PCR–RFLP) method. Amplification was performed in MJ-Mini Thermal cycler (Bio-Rad, Tokyo, Japan).

Genotyping of rs-7668258 (161 C>T) was carried out using the forward primer, 5′-GAT​CTG​TCA​CTG​CTA​CTG​TTC-3′ and reverse primer 5′-GTC​TGA​GCA​TGT​GGA​TGG​CCA-3′, with annealing temperature being 59°C and BstNI was the restriction enzyme (RE) used. For the genotyping of rs-12233719 (211 G>T).

Primers were F: 5′-TGC​TTT​AGC​TCT​GGG​AAT​TGT -3′ R: 5′-TGC​ATG​ATG​AAA​TTC​TCC​AAC-3´, RE MbiI, annealing temperature 59°C.

Primers used for rs-7662029 (268 A>G) were F: 5′-TCC​AAC​TGA​TTG​TTA​TGG​TAG​AT-3′

R: 5′-GCT​GTT​CCT​TTC​TGT​CAT​TTC​TC-3′ at an annealing temperature of 54°C, RE BglII.

The primers were obtained from Bionova Suppliers, chemicals required for DNA isolation and other procedures were procured from Merck and Sigma laboratory (Analytical grade). PCR was carried out with an initial denaturation enzyme activation step at 95°C for 5 min, amplification step for 35 cycles at 95°C for 30 s, annealing temperature for 30 s, treatment at 72°C for 30 s, final extension step at 72°C for 5 min. The amplified product of DNA was confirmed on a 2% agarose gel with ethidium bromide.

Amplified PCR product was digested with respective restriction enzymes (BstNI, MbiI, BglII) respectively for UGT2B7 (C161T, G211T, A268G) genes. The restricted fragment was separated on a 3% agarose gel with ethidium bromide. The genotype was assigned based on the results of the analysis of the digestion patterns. Trough level serum concentration of Sodium valproate was measured by HPLC analysis (Agillent Technologies, 1,260 Infinity). Standard Sodium valproate was procured from sigma laboratory for HPLC estimation.

Parents were asked to maintain diary in which seizure attack, duration of the attack, and any changes in the pattern from the prior episode, untoward response (loss of appetite, nausea, vomiting, discoloration of eye, pain abdomen, rashes) to the treatment at any point of time and missed medication dates to be mentioned. Were mentioned and shared with the treating pediatrician. Any of the above untoward effects and missing more than two doses of medication in a month was considered non-compliance.

The clinical outcome was evaluated in terms of sodium valproate efficacy and safety. The drug’s efficacy was assessed in terms of treatment responders and non-responders. Patients who had no recurrence or only two bouts of seizure attack in 6 months of follow-up were considered responders and sodium valproate monotherapy was well tolerated with no side effects. Non-responders were defined as individuals who had two or more seizure attacks in 6 months or experienced any adverse drug effect after starting therapy (Carpay and Arts, 1996). Tolerance was determined by comparing biochemical markers at baseline and 6 months later. If any patients developed jaundice, pancreatitis, or showed symptoms of thrombocytopenia, their tolerability was examined.

### Calculating the sample size

Taking SD of drug concentration as 23 μg/ml from the referred article [75] and fixing the margin of error = 5, the sample size would be 82. Z1-α/2 2 n = E x S2 n = 1.96 two x (23)2 5 n = 82 Considering 15% as attrition, the calculated total sample size is 95. The sample size calculated was 82. Expecting an attrition of 15%, the final sample size was 95. However because of COVID-19 pandemic we could obtain all the parameters of 75 patients only.

### Statistical analysis

The obtained data was reported as frequency, percentage for qualitative data and mean and SD for quantitative data Using SPSS software version 16.0 statistical analysis was carried out. The Chi square test was used to find the association of UGT 2B7 and clinical responsiveness. A paired 't' test was used to compare the biochemical markers between baseline and 6 months follow-up in different age groups. Statistical significance was defined as a *p* value of less than 0.05.

D′ and r2 were calculated to determine the extent of LD in paired combinations of SNPs. The SHESIS plus and SNPstat online programme platforms were used to assess the LD between UGT2B7 haplotypes (C161T, A268G, and G211T). The analysis excluded haplotypes with frequencies of less than 0.03.

## Results

**FIGURE 1:**
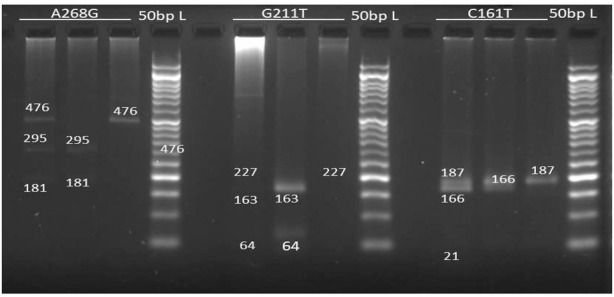
Electrophoresis gel image patterns of UGT2B7 (A268G, G211T, C161T) gene after digestion with *BglII, MbiI, BstNI* enzymes respectively (L): 50bp Ladder, Lane 1): homozygosity for variant allele, Lane(2): homozygosity for common allele, Lane 3): heterozygosity.

The mean age of our patients was 8.5 ± 4.3 years (2.2–17.3 years), and the average BMI was 16.5 ± 4.3 (7.81–32.84). The study subjects included 57 boys and 43 females. Between baseline and 1 year, there was a significant difference in sodium valproate concentration, adjusted sodium valproate concentration, creatinine, and platelet values. At different time intervals, there was no significant variation in hepatic and renal function test parameters. In UGT2B7 (C161T) gene, CC (38 percent), CT (43 percent), TT (19 percent) pattern was detected, AA (15 percent), AG (39 percent), GG (46 percent) in (A268G) gene, and GG (80 percent), GT (18 percent), TT (02 percent) in (G211T) gene (Figure 1).

**FIGURE 2:**
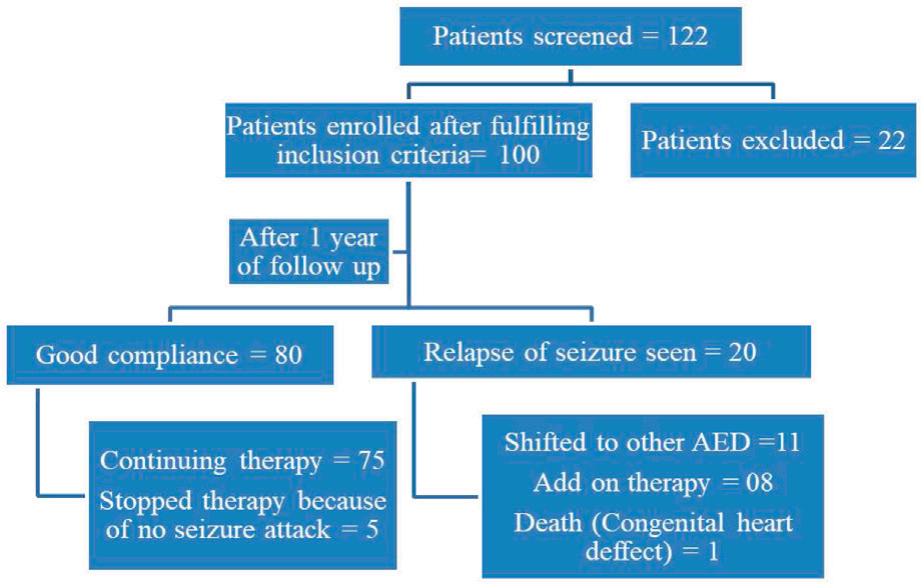
STROBE Flow chart of patient recruitment and follow-up details.

Frequencies of alleles and chi-square values of Hardy-Weinberg equilibrium are represented in (Table 1). After anti-epileptic drug therapy, the clinical outcome (seizure frequency) was monitored at 6-months and 1-year intervals (Figure 2). Responders (total seizure control from the day of enrolment to the day of assessment) and non-responders (two episodes of seizures from the day of enrollment) were compared.

**TABLE 1:** Hardy Weinberg Equilibrium for UGT2B7 gene.

Gene variant		Frequency of pattern in UGT2B7 gene		
		C161T	A268G	G211T
Common homozygotes	Observed	38	15	80
	Expected	35.40	11.90	79.21
**Heterozygotes**	**Observed**	**43**	**39**	**18**
	Expected	48.19	45.19	19.58
**Rare Homozygotes**	**Observed**	**19**	**46**	**02**
	Expected	16.40	42.90	1.21
**Chi-square value**		1.619	1.879	0.651

∗*p* ≤ 0.05, df = 4, wpcalc online calculator used.

The G211T gene polymorphism in the UGT2B7 gene exhibited a lower frequency of mutant carrier allele type than the wild type. The mutant carrier allele types C161T and A268G were found to be more common. Eighty patients (80%) reacted effectively to the treatment with no seizure relapses, while 20 patients (20%) experienced a seizure relapse with two or more occurrences. Other anti-epileptic medicines were prescribed for these patients. There was no statistically significant association between UGT2B7 (C161T, A268G, G211T) gene polymorphism and clinical efficacy (seizure control) (Table 2). Eighty-three patients (83%) had generalised tonic-clonic seizures (GTCS), while 17 patients (17%) had complex partial seizures (CPS).

**TABLE 2:** Association of UGT2B7 gene polymorphisms with Clinical Outcome (N = 100).

Genotype UGT2B7	Polymorphism pattern	Polymorphism in percentage (%)	Clinical outcome		*p*-value
			Responders (N = 80)	Non- responders (N = 20)	
C161T	Wild	37	30	07	0.526
	Mutant	63	50	13	
A268G	Wild	15	13	02	0.382
	Mutant	85	67	18	
G211T	Wild	80	65	15	0.365
	Mutant	20	15	05	

Statistical test used is Chi Square test.

There was no statistically significant association found between seizure type UGT2B7 (C161T, A268G, G211T) gene polymorphism and seizure type (Table 3).

**TABLE 3:** Association of UGT2B7 gene polymorphisms with Seizure type (N = 100).

GenotypeUGT2B7	Polymorphism pattern	Polymorphism in percentage (%)	Seizure type		*p*-value
			GTCS (N = 83)	CPS(N = 17)	
C161T	Wild	37	28	09	0.112
	Mutant	63	55	08	
A268G	Wild	15	12	03	0.491
	Mutant	85	71	14	
G211T	Wild	80	65	15	0.286
	Mutant	20	18	02	

Statistical test used is Chi-Square test.

During the baseline, 6-month, and 1-year follow-ups, all of the children had normal liver function values and renal function parameters (Table 4). Between baseline and 1 year, there was a significant difference in sodium valproate concentration, adjusted sodium valproate concentration, creatinine, and platelet values. At different time intervals, there was no significant variation in hepatic and renal function test parameters (Table 4).

**TABLE 4:** Comparison of biochemical and hematological parameters at different time intervals.

Characteristics	Basal (N = 100) (mean ± SD/range)	6 Months (N = 75) (mean ± SD)	1 Year (N = 72) (mean ± SD)	*p*-value
Age (in years)	8.5 (2.2–17.3)			
Sex M: F	57: 43			
BMI	16.5 ± 4.3	16.6 ± 3.7	17.34 ± 3.6	0.000*
Sodium Valproate Concentration (µmol/L)	715.86 ± 236.31	640.74 ± 291.753	527.58 ± 239.08	0.000*
Albumin (g/L)	44.4 ± 3.4	44.3 ± 3.4	44.3 ± 3.5	0.502
Total Protein (g/L)	73.4 ± 6.3	75 ± 6.1	74.9 ± 7.1	0.257
SGOT (IU/L)	31.18 ± 11.2	27.24 ± 8.30	28.86 ± 8.36	0.670
SGPT (IU/L)	13.80 ± 6.12	12.60 ± 4.88	12.39 ± 5.26	0.415
Alkaline Phosphate (IU/L)	192.5 ± 72.6	203.9 ± 79.9	206.2 ± 80.1	0.622
Direct Bilirubin (μmol/L)	1.881 ± 1.71	1.71 ± 0.855	1.881 ± 1.368	0.767
Total Bilirubin (μmol/L)	5.13 ± 3.42	4.959 ± 2.565	5.13 ± 3.078	0.880
Blood Urea (mmol/L)	7.511 ± 2.684	6.865 ± 2.598	7.08 ± 2.356	0.335
Serum Creatinine (μmol/L)	42.43 ± 18.56	34.47 ± 11.92	34.47 ± 10.60	0.000*
Amylase (IU/L)	85.13 ± 43.1	79.70 ± 32.8	77.59 ± 32.8	0.234
Platelets (cells/mm^3^)	282,480 ± 64,738	277,330 ± 42,979	295,850 ± 30,500	0.000∗

One-way ANOVA, followed by Bonferroni’s post hoc test.

∗*p*-value <0.05 was statistically significant.

During baseline and 1-year follow-up, all children exhibited normal liver function, hematological, and renal function parameters, with the exception of two cases. Acute pancreatitis developed in one patient, who had increased serum lipase and USG findings indicative of pancreatitis. This patient was given de-challenge, and he recovered totally within a week. Levetiracetam was prescribed for the patient. The Naranjo algorithm was used to assess causality, and a score of +6 was obtained, indicating that ADR was most likely caused by sodium valproate. The use of sodium valproate has been associated to the development of acute pancreatitis.

Analysis of combined genotyping data for patients and controls was used to look for LD between pairs of SNPs. Controls were paediatric epileptics who reacted to VPA monotherapy, whereas non-responders were considered cases. The global statistic R2 was created, as well as the statistic D′, which accounts for the constraints on R imposed by differing allele frequencies of the marker pair.

The three SNPs of UGT2B7 showed a weak LD between A268G and G211T of UGT2B7, as suggested by low D′ values, 0.3, 0.73, and 0.35 between three alleles. Low *R* ^2^ values (0.06,0.03 and 0.01 respectively) do not support the co-inheritance of the above alleles. Low *R* ^2^ values observed could be due to the low allele frequencies. Closer the D’ and *R* ^2^ values to 1, a stronger disequilibrium between the alleles is suggested which imply strong chance of co-inheritance of the alleles. If the values are closer or equal to zero, it is suggestive of independence of alleles. None of the gene interactions and their association with the response to therapy were statistically significant as shown by binary analysis (Tables 5, 6). However the association of the interactions of **C161T and G211T** with the response to therapy were significant (*p* = 0.014) (Table 7).

**TABLE 5:** Haplotype association of UGT2B7 C161T and A268G with response (n = 100, adjusted by Age + Sex).

	C161T	A268G	Freq	Or (95%CI)	*p*-value
1	C	G	0.3975	1.00	---
2	T	G	0.2575	0.76 (0.33–1.77)	0.53
3	T	A	0.1825	0.84 (0.29–2.41)	0.75
4	C	A	0.1625	0.41 (0.11–1.54)	0.19
Global haplotype association *p*-value: 0.5					

**TABLE 6:** Haplotype association of UGT2B7 A268G and G211T with response (n = 100, adjusted by Age + Sex).

	A268G	G211T	Freq	Or (95%CI)	*p*-value
1	G	G	0.5635	1.00	---
2	A	G	0.3265	0.79 (0.36–1.70)	0.54
3	G	T	0.0915	1.94 (0.69–5.50)	0.21
4	A	T	0.0185	0.00 (-Inf - Inf)	1
Global haplotype association *p*-value: 0.29					

**TABLE 7:** Haplotype association of UGT2B7 C161T and G211T with response (n = 100, adjusted by Age + Sex).

	C161T	G211T	Freq	Or (95%CI)	*p*-value
1	C	G	0.5145	1.00	---
2	T	G	0.3755	1.69 (0.76–3.76)	0.21
3	T	T	0.0645	0.48 (0.06–3.74)	0.49
4	C	T	0.0455	14.15 (2.44–82.24)	0.004
Global haplotype association *p*-value: 0.014					

A haplotype analysis of all the genes was performed using haplotype frequencies predicted by the Shesisplus for association with the response to therapy. Of the pairwise haplotypes association, none were statistically significant except for a haplotype TAACGT, with a chi square value of 7.77, *p* = 0.005, with OR 0.121 [95% CI = 0.021–0.689]. This suggests a good response to therapy among these haplotypes.

## Discussion

The association between UGT2B7 gene patterns and clinical outcomes were investigated in this study. Interindividual variability in pharmacokinetics and pharmacodynamics in paediatric epileptic patients on sodium valproate monotherapy may be influenced by gene polymorphism. Many researchers have looked into the effect of gene polymorphisms on sodium valproate pharmacokinetics and pharmacodynamics. When compared to the Chinese and Japanese populations, the pattern of UGT2B7 gene polymorphism was similar for UGT2B7 (C161T, A268G, G211T type) (Table 8) (Hung et al., 2011; Ma et al., 2013; Inoue et al., 2014; Yin-xiang et al., 2015; Du et al., 2016). The prevalent genetic polymorphism of UGT2B7 (C161T) pattern reported in Chinese paediatric epileptic population (Hung et al., 2011) was wild type, whereas the predominant pattern of UGT2B7 (C161T) gene observed in our and Japanese study populations was mutant carrier allele type. However, no genetic differences between our patients and Japanese pediatric epileptic patients were found (Inoue et al., 2014).

**TABLE 8:** Details of prevalence of UGT2B7 gene polymorphisms in various populations.

Genotype UGT2B7	Chinese patients with epilepsy				Japanese	Current study
	Hongying et al. (n = 162)	Zhongliang et al. (n = 102)	Hung et al. (n = 162)	Sun et al. (n = 102)	Inoue et al. (n = 78)	(n = 100)
C161T						
CC	NS	NS	55.56%	NS	37.2%	38.4%
CT	NS	NS	37.04%	NS	53.8%	42.4%
TT	NS	NS	07.41%	NS	09.0%	19.2%
A268G						
AA	05.65%	05.88%	NS	NS	NS	15.2%
AG	48.79%	49.01%	NS	NS	NS	39.4%
GG	45.56%	45.11%	NS	NS	NS	45.4%
G211T						
GG	59.27%	72.55%	NS	76.47%	NS	80.8%
GT	36.29%	25.50%	NS	22.55%	NS	17.2%
TT	04.44%	01.95%	NS	0.98%	NS	02.0%

NS, not studied.

Several investigations in Chinese juvenile epileptics found that UGT2B7 (A268G) had a greater population of mutant carrier allele type than wild type, while UGT2B7 (G211T) had a lower population of mutant carrier allele than wild type.

To date, research on the impact of UGT2B7 polymorphism on substrate drug metabolism have been conflicting. Guo et al. (Guo et al., 2012) found that polymorphisms had no effect on serum valproate concentrations. UGT2B7 (A268G) plays a significant function in sodium valproate metabolism, according to Hongying et al. and Zhongliang et al. (Ma et al., 2013; Du et al., 2016).

Patients with the UGT2B7 (C161T) genotype had lower adjusted plasma valproic acid concentrations than those with the mutant carrier allele type, according to Inoue et al. (Inoue et al., 2014). To yet, none of the existing literatures have described the impact of UGT genetic variations on sodium valproate monotherapy clinical outcomes. Hung et al. tentatively tackled it, explaining that patients with strong medication response (seizure free/good seizure management) were evaluated for numerous genetic influences on VPA pharmacokinetics, including the UGT1A6 gene (Hung et al., 2011). However, we found no association between distinct polymorphism patterns of UGT2B7 and clinical efficacy or tolerability in our research.

Our study has a limitation due to the small sample size. Only a metacentric investigation including multiple regions could explain our limitation and add quality to the data in terms of genetic pattern in the current context of modified protocol in the treatment of pediatric epilepsy (preference of levetiracetam over sodium valproate).

## Conclusion

Our findings showed that varied patterns of UGT2B7 genetic polymorphisms have no effect on the efficacy and tolerability of sodium valproate in the treatment of epilepsy. We also came to the conclusion that sodium valproate was well tolerated by pediatric epilepsy patients and might be employed as an effective anti-epileptic drug.

## Data Availability

The original contributions presented in the study are included in the article/Supplementary Materials, further inquiries can be directed to the corresponding author.
